# Genetic Variants Associated with Elevated Plasma Ceramides in Individuals with Metabolic Syndrome

**DOI:** 10.3390/genes13081497

**Published:** 2022-08-22

**Authors:** Sanghoo Lee, Seol-A Kim, Yejin Kim, Juhoon Kim, Gayeon Hong, Jeonghoon Hong, Kyeonghwan Choi, Chun-Sick Eom, Saeyun Baik, Mi-Kyeong Lee, Kyoung-Ryul Lee

**Affiliations:** 1 Center for Companion Biomarker, Seoul Clinical Laboratories Healthcare Inc., Yongin 16954, Gyeonggi-do, Korea; 2 Center for Health Check-Up, HANARO Medical Foundation, Seoul 03159, Korea; 3 Central Laboratory, Seoul Clinical Laboratories Healthcare Inc., Yongin 16954, Gyeonggi-do, Korea; 4 Department of MyGenome, Seoul Clinical Laboratories, Yongin 16954, Gyeonggi-do, Korea

**Keywords:** metabolic syndrome, ceramide species, plasma ceramide accumulation, genetic determinants, SNP genotyping, KoreanChip

## Abstract

Metabolic syndrome (MetS) is a complex condition of metabolic disorders and shows a steady onset globally. Ceramides are known as intracellular signaling molecules that influence key metabolism through various pathways such as MetS and insulin resistance. Therefore, it is important to identify novel genetic factors related to increased plasma ceramides in subjects with MetS. Here we first measured plasma ceramides levels in 37 subjects with MetS and in 38 healthy subjects by ultra-performance liquid chromatography-tandem mass spectrometry (UPLC-MS/MS). Specifically, levels of C16 ceramide (Cer-16), C18 ceramide (Cer-18), C20 ceramide (Cer-20), C18 dihydroceramide (DhCer-18), C24 dihydroceramide (DhCer-24), and C24:1 dihydroceramide (DhCer-24:1) were significantly increased in MetS group (*p* < 5.0 × 10^−2^). We then performed single nucleotide polymorphism (SNP) genotyping to identify variants associated with elevated plasma ceramides in MetS group using Axiom^®^ Korea Biobank Array v1.1 chip. We also performed linear regression analysis on genetic variants involved in ceramide synthesis and significantly elevated plasma ceramides and dihydroceramides. Ten variants (rs75397325, rs4246316, rs80165332, rs62106618, rs12358192, rs11006229, rs10826014, rs149162405, rs6109681, and rs3906631) across six genes (*ACER1*, *CERS3*, *CERS6*, *SGMS1*, *SPTLC2*, and *SPTLC3*) functionally involved in ceramide biosynthesis showed significant associations with the elevated levels of at least one of the ceramide species in MetS group at a statistically significant threshold of false discovery rate (FDR)-adjusted *p* < 5.0 × 10^−2^. Our findings suggest that the variants may be genetic determinants associated with increased plasma ceramides in individuals with MetS.

## 1. Introduction

MetS is defined as a cluster of metabolic disorders including high blood glucose, hypertension, abdominal obesity, dyslipidemia, and insulin resistance that increase the risk of cardiovascular diseases and type 2 diabetes. MetS is characterized by a disorder of energy utilization and storage [1]. In particular, the proportion of subjects with MetS continues to increase due to obesity trends, sedentary lifestyle, and high consumption of calories [2,3,4], which makes MetS one of the major global medical concerns expected to record a steadily high incidence rate [5].

Fatty acids are stored primarily as triacylglycerols (TGs) in subcutaneous adipose tissue [6]. Hydrolysis of TGs from adipocytes results in an increase in plasma long-chain fatty acid content and subsequent ectopic lipid accumulation [7]. This lipid overload causes problems such as metabolic diseases, especially when lipid substances such as ceramide are accumulated.

Ceramides are a homogenous class of sphingolipids, as signaling molecules, which play important roles in cellular stress, inflammation, and apoptosis [8,9]. The sphingolipids are also one of the hallmarks of metabolic disorders [10,11]. Sphingolipids are a class of lipids containing a backbone of sphingoid bases, a set of aliphatic amino alcohols, including sphingosine. Dysfunction in ceramide synthesis is reported to cause ceramide accumulation, which interferes with insulin signal transduction and is responsible for the development of insulin resistance and MetS [12]. Thus, it is clinically important to identify the factors that cause ceramide accumulation in blood. Moreover, given their many important functions in human disease, it is also essential to measure the concentration of these sphingolipid molecules in the blood using a sensitive and accurate method.

Recently, genetic variants related to clinical features that influence the development of MetS have been identified by sequencing approaches such as microarray chip [13,14] and next-generation sequencing (NGS) [15,16].

According to a MetS study with Korean twins, various factors affecting MetS have very high heritability [17]. Additionally, a recent study in European populations has reported that circulating sphingolipids concentrations are under strong genetic control [18]. Thus, we designed the study to identify Korean-specific SNP markers associated with ceramide accumulation in blood of subjects with MetS. Herein, we produced genotype data using the Korean-specific SNP genotyping microarray chip, known as the Korea Biobank Array 1.1 or KoreanChip [19], which was originally designed by Center for Genome Science, Korea National Institute of Health, Korea.

In this study, we measured plasma ceramide levels in both subjects with MetS and healthy controls using UPLC-MS/MS and then identified genetic variants associated with plasma ceramide accumulation in subjects with MetS using the KoreanChip.

## 2. Materials and Methods

### 2.1. Study Participants and Their Classification

Thirty-seven participants were enrolled as the MetS group through clinical classification based on the results of blood biochemical tests and health examinations, and 38 participants with no clinical features of MetS were enrolled as the healthy control group (Table 1). All study participants were recruited at the health check-up center of Hanaro Medical Foundation, Seoul, Korea. The 37 individuals with MetS were selected according to a harmonized definition of International Diabetes Federation/National Heart, Lung, and Blood Institute/American Heart Association/International Association for the Study of Obesity [20]. The subjects with MetS had at least three clinical features among hyperglycemia (fasting glucose: ≥100 mg/dL), decreased HDL-cholesterol (HDL-C) (<40 mg/dL in men; <50 mg/dL in women), increased TG (≥150 mg/dL), hypertension (≥130 mmHg systolic and/or ≥85 mmHg diastolic), and central obesity (waist circumference >90 cm in men; >85 cm in women) that followed a definition of the Korean Academy of Family Medicine [21]. Subjects with chemotherapy, two or more combination medications, or anticancer drugs on special medications were excluded from this study.

The study protocol was approved by the Institutional Review Board of Seoul Clinical Laboratory (SCL IRB-2019-015), which complies with the principles of the Declaration of Helsinki. Written informed consent was obtained from all study participants.

### 2.2. Selection of Ceramides and Dihydroceramides

Among sphingolipids, we targeted three representative ceramides (C16 ceramide (d18:1/16:0) (Cer-16), C18 ceramide (d18:1/18:0) (Cer-18), and C20 ceramide (d18:1/20:0) (Cer-20)) and four representative dihydroceramides (C16 dihydroceramide (d18:0/16:0) (DhCer-16), C18 dihydroceramide (d18:0/18:0) (DhCer-18), C24 dihydroceramide (d18:0/24:0) (DhCer-24), and C24:1 dihydroceramide (d18:0/24:1) (DhCer-24:1)) based on previously reported result [22]. Then, we investigated whether the concentrations of these ceramides and dihydroceramides were changed in subjects with MetS.

### 2.3. Sample Preparation for LC-MS/MS Analysis

Cer-16, Cer-18, Cer-20, C18 ceramide-*d*_7_ (d18:1-*d*_7_/18:0) (Cer-18-*d*_7_), DhCer-16, DhCer-18, DhCer-24, DhCer-24:1, C13 dihydroceramide-*d*_7_ (d18:0-*d*_7_/13:0) (DhCer-13-*d*_7_) were purchased from Avanti Polar Lipids (Alabaster, AL, USA). Formic acid, trifluoroacetic acid, chloroform, and bovine serum albumin (BSA) were obtained from Sigma-Aldrich (Darmstadt, Germany). All HPLC solvents were HPLC grade, which were purchased from Thermo Fisher Scientific (Waltham, MA, USA).

All stock solutions (1 mg/mL) were prepared in methanol/chloroform (2:1, *v*/*v*). Working solution for calibration curve and spiking in solution containing Cer-16, Cer-18, Cer-20, DhCer-16, DhCer-18, DhCer-24, and DhCer-24:1 was prepared by serial dilution. Internal standard (ISTD) solution containing 100 ng/mL Cer-18-*d*_7_ and 100 ng/mL DhCer-13-*d*_7_ was prepared in methanol/chloroform (2:1, *v*/*v*).

Plasma was separated from each 1 mL of whole bloods from study participants by centrifugation at 400 *g* for 15 min and stored at −80 °C before UPLC-MS/MS analysis. Plasma sample of 50 μL was subjected to liquid–liquid extraction with 2 mL chloroform-methanol (1:2, *v*/*v*) added with trifluoroacetic acid and spiked with the ISTD solution to reach a final volume of 0.1%. Then, 1.5 mL chloroform and 0.5 mL water were added to each extract and vortexed, following which each sample was centrifuged at 3220 *g* for 5 min at 4 °C. The lower organic phase (2 mL) was evaporated at 40 °C under a gentle stream of nitrogen and the residues were reconstituted in 0.1 mL methanol-chloroform (9:1, *v*/*v*). After further mixing and centrifugation, the samples were transferred to vials and analyzed by UPLC-MS/MS.

### 2.4. LC-MS/MS Analysis of Plasma Ceramide Species

In this study, UPLC-MS/MS analysis was performed with a modification of the method by Basit and coworkers [22]. UPLC–MS/MS analysis was carried out on a SCIEX QTRAP^®^ 4000 mass spectrometer coupled to an Agilent 1290 Infinity LC system. Chromatographic separation was achieved using a BEH C18 column (2.1 × 50 mm, 1.7 μm) eluted at a flow rate of 0.4 mL/min. The mobile phase consisted of 0.1% formic acid in acetonitrile/water (20:80, *v*/*v*) (solvent A) and 0.1% formic acid in acetonitrile/2-propanol (20:80, *v*/*v*) (solvent B). A step gradient program was developed for optimal separation of all ceramide species as follows: 0.0–1.0 min 75% solvent B, 1.0–9.0 min 75 to 83% solvent B, 9.0–9.1 min 83 to 100% solvent B, 9.1–10.0 min 100% solvent B. The column was then reconditioned to 75% solvent B for 2.0 min. The total run time for analysis was 12 min and the injection volume was 2 µL. The LC-MS/MS system was operated in positive ion mode electrospray ionization (ESI). Quantification of ceramide and dihydroceramide species was performed using multiple-reaction monitoring (MRM) of the transitions of mass-to-charge ratio (*m*/*z*) 538.50 → 264.20, *m*/*z* 566.50 → 264.20, *m*/*z* 594.50 → 264.20, *m*/*z* 540.45 → 522.65, *m*/*z* 568.54 → 550.54, *m*/*z* 652.51 → 634.80, *m*/*z* 650.49 → 632.80, *m*/*z* 573.50 → 271.30, and *m*/*z* 505.46 → 487.68 for Cer-16, Cer-18, Cer-20, DhCer-16, DhCer-18, DhCer-24, DhCer-24:1, Cer-18-*d*_7_, and DhCer-13-*d*_7_, respectively. The following parameters were set on the MS/MS system: curtain gas, 25 psi; ion spray voltage, 5500 V; temperature, 550 °C; gas 1 and gas 2, 50 and 60 psi, respectively.

Calibration curves were constructed by plotting the corresponding peak area ratios of analyte/internal standard versus the corresponding analyte concentrations using weighted (1/*x*^2^) least squares regression analysis. Results are expressed as mean ± S.D. using GraphPad Prism (Ver. 8.2.0). Significance was evaluated by paired *t*-test. *p* < 5.0 × 10^−2^ were considered significant statistically. Coefficients of correlation (*r*^2^) between plasma ceramide level of subjects with MetS and their clinical indicators were calculated using MedCal^®^ software (Ver. 20.014).

### 2.5. SNP Genotyping

DNA samples were extracted from 200 μL of dispensed whole blood using a Roche MagNA Pure 96 System with Roche MagNa Pure 96 DNA and Viral NA Small Volume Kit. Two hundred microliters of DNA samples were genotyped following the manufacturer’s instructions on a ThermoFisher GeneTitan™ Multi-Channel Instrument with the Axiom^®^ Korea Biobank Array v1.1 chip. Genotype result CEL files were transformed into a PLINK input format file (ped format) using the Axiom^®^ Analysis Suite program. Samples not reaching the cut-off criteria, Dish QC (DQC) ≥0.82 and call rate ≥0.97, were re-genotyped using another KoreanChip. Genotyped files were analyzed using PLINK (Ver. 1.90) [23]. SNPs classified as Mendelian inconsistencies (--mendel-multigen) were regarded as missing and SNPs with low genotyping rates (<0.05), low minor allele frequency (<0.01), and outlying heterozygosity (<0.31 or >0.33) were excluded.

### 2.6. Association Analysis

Significantly associated SNPs were analyzed using Ensembl API Client (Ver. 1.1.5) with genome assembly hg19 to annotate gene symbols. Annotated SNPs included in the ceramide biosynthesis genes and significant SNPs with *p* values of <5.0 × 10^−2^ in Hardy-Weinberg equilibrium analysis were selected.

Selected SNPs were analyzed for association with plasma dihydroceramide and ceramide levels using linear regression models. Then, the results were adjusted to elucidate false positives resulting from multiple testing by Benjamini–Hochberg FDR-adjusted *p* value calculation and associations were considered statistically significant at FDR-adjusted *p* values of <5.0 × 10^−2^. Association analyses were undertaken for each dihydroceramide and ceramide using the R package software (Ver. 3.5.3).

To identify if the SNPs were in linkage disequilibrium (LD) with the genes involved in sphingolipid biosynthesis in the HapMap CEPH Utah residents with ancestry from northern and western Europe (CEU), Han Chinese in Beijing (CHB), and Japanese in Tokyo, Japan (JPT) data, LD analysis was performed using LDmatrix software (https://analysistools.cancer.gov/LDlink/?tab=idmatrix, accessed on 1 July 2022).

## 3. Results

### 3.1. Clinical Characteristics of Study Participants

The baseline clinical indicator of 38 healthy controls and the 37 subjects with MetS are shown in Table 1. As a result of analyzing the clinical indicators of all participants, all indicators except for total cholesterol showed statistically significant differences between MetS and control (*p* < 5.0 × 10^−2^). Especially, leptin, adiponectin, hs-CRP, insulin, and HOMA-IR showed significant differences between the two groups.

### 3.2. LC-MS/MS Measurement of Plasma Level of Ceramides

**Figure 1:**
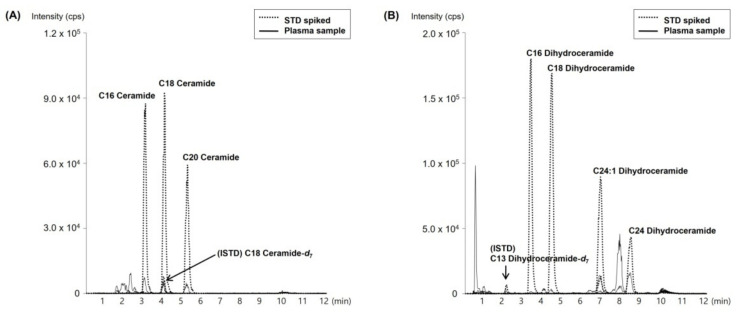
MRM chromatograms of (**A**) Cer-16, Cer-18, and Cer-20, and (**B**) DhCer-16, DhCer-18, DhCer-24, and DhCer-24:1 spiked at 50 ng/mL in 4% BSA with control human plasma. The STD and ISTD indicate the standard and internal standard, respectively. Cer-18-*d*_7_ and DhCer-13-*d*_7_ were used as ISTDs.

From the separated plasma samples, the levels of each of the seven types of ceramides (Cer-16, Cer-18, Cer-20, DhCer-16, DhCer-18, DhCer-24, and DhCer-24:1) were measured. Under optimal UPLC-MS/MS analysis conditions, the ceramide species were clearly separated in a single 12 min long UPLC-MS/MS run (Figure 1). Overall, plasma ceramide levels except for DhCer-16 level were significantly elevated in the subjects with MetS compared to the healthy controls (*p* < 5.0 × 10^−2^) (Table 2). 

### 3.3. Identification of SNPs Associated with Ceramide Biosynthesis Pathway

**Figure 2:**
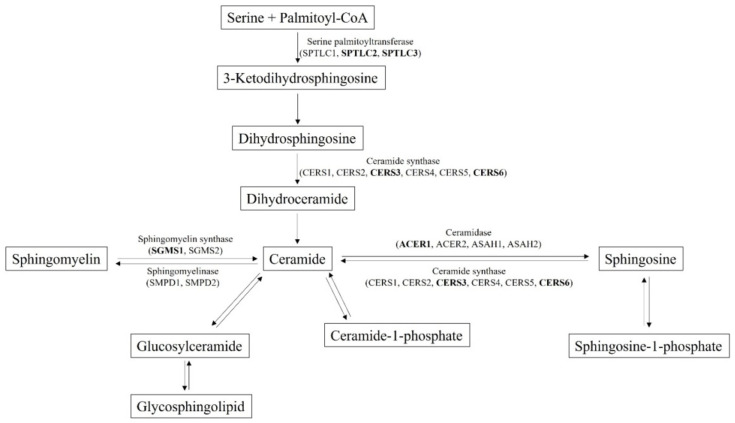
Genes associated with ceramide biosynthesis pathway. Loci in bold are those with variants identified in our study.

The six significantly elevated plasma ceramide levels in subjects with MetS were considered for association analysis with selected SNPs (*p* < 5.0 × 10^−2^) genotyped using the KoreanChip. From SNPs that passed the threshold of *p* < 5.0 × 10^−2^ in the MetS group compared to that of the control group, we narrowed down the number of candidate SNPs by gene function annotation. Genes known to be associated with the ceramide synthesis pathway (Figure 2) were regarded as target genes and SNPs from these genes were selected.

Correlation analysis between plasma ceramide levels measured by UPLC-MS/MS and the selected SNPs was performed using a linear regression model from the R package. 

**Figure 3:**
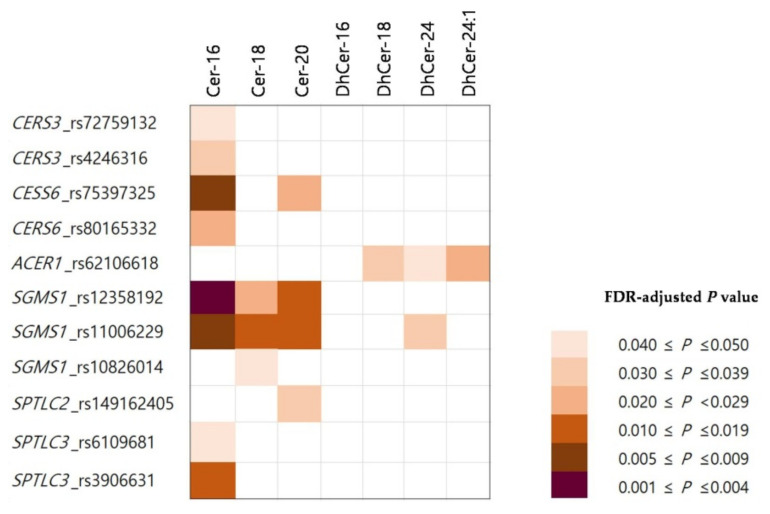
A plot of correlation between SNPs and plasma ceramides.

A total of 10 SNPs (rs75397325, rs4246316, rs80165332, rs62106618, rs12358192, rs11006229, rs10826014, rs149162405, rs6109681, and rs3906631) found in six genes (*CERS3*, *CERS6*, *ACER1*, *SGMS1*, *SPTLC2*, and *SPTLC3*) showed associations with elevated plasma levels of at least one of the ceramide types in MetS group (FDR-adjusted *p* values <5.0 × 10^−2^) (Table 3). The plot of correlation between SNPs and plasma ceramides is also shown in Figure 3. 

The *CERS6* located at chromosome 2 encodes ceramide synthase [24]. The two SNPs rs75397325 and rs80165332 at this locus showed statistically significant associations with elevated plasma Cer-16 (FDR-adjusted *p* = 5.2 × 10^−3^ and *p* = 2.8 × 10^−2^, respectively). The SNP rs75397325 at this locus also showed association with elevated plasma Cer-20 (FDR-adjusted *p* = 2.1 × 10^−2^). The SNP rs4246316 in *CERS3* located at chromosome 15 exhibited association with elevated plasma Cer-16 (FDR-adjusted *p* = 3.1 × 10^−2^). However, the SNP rs72759132 in *CERS3* did not show association with elevated Cer-16 after FDR correction (FDR-adjusted *p* = 5.0 × 10^−2^).

The *ACER1* located at chromosome 19 encodes alkaline ceramidase [25]. The SNPs rs62106618 at this locus showed association with elevated plasma DhCer-18 (FDR-adjusted *p* = 3.4 × 10^−2^), DhCer-24 (FDR-adjusted *p* = 4.7 × 10^−2^), and DhCer-24:1 (FDR-adjusted *p* = 2.6 × 10^−2^), respectively. 

The *SGMS1* located at chromosome 10 encodes sphingomyelin synthase [26]. The three SNPs at this locus exhibited associations with increased dihydroceramide or ceramide species in plasma. The SNPs rs12358192 at this locus showed association with elevated plasma Cer-16 (FDR-adjusted *p* = 2.6 × 10^−3^), Cer-18 (FDR-adjusted *p* = 2.3 × 10^−2^), and Cer-20 (FDR-adjusted *p* = 1.5 × 10^−2^), respectively. The SNP rs11006229 in *SGMS1* was associated with elevated plasma DhCer-24 (FDR-adjusted *p* = 3.9 × 10^−2^), Cer-16 (FDR-adjusted *p* = 7.8 × 10^−3^), Cer-18 (FDR-adjusted *p* = 1.8 × 10^−2^), and Cer-20 (FDR-adjusted *p* = 1.0 × 10^−2^), respectively. The SNP rs10826014 was also associated with Cer-18 after FDR correction (FDR-adjusted *p* = 4.2 × 10^−2^). The *SPTLC2* and *SPTLC3* located at chromosome 14 and 20, respectively, encode serine palmitoyltransferase long chain base [18]. The SNP rs149162405 in *SPTLC2* and the two SNPs rs6109681 and rs3906631 in *SPTLC3* exhibited associations with Cer-20 (FDR-adjusted *p* = 3.6 × 10^−2^), Cer-16 (FDR-adjusted *p* = 4.4 × 10^−2^), and Cer-16 (FDR-adjusted *p* = 1.3 × 10^−2^), respectively.

We performed LD analysis of the SNPs located within *CERS3*, *CERS6*, *SGMS1*, and *SPTLC3* genes involved in sphingolipid biosynthesis in the HapMap CEU data. The LD analysis of the three SNPs located within *SGMS1* revealed that moderate LD was observed between rs12358192 and rs11006229 (*r*^2^ = 0.292), whereas weak LD between rs11006229 and rs10826014 and between rs10826014 and rs12358192 (both *r*^2^ < 0.01) (Supplementary Table S1). Weak LD was also observed between rs72759132 and rs4246316 located within *CERS3* and between rs6109681 and rs3906631 within *SPTLC3* (both *r*^2^ < 0.20). There was no LD between the two SNPs (rs80165332 and rs75397325) located within *CERS6*. We also performed LD analysis of the SNPs in the HapMap CHB and JPT data. Interestingly, the LD analysis revealed that rs12358192 and rs11006229 located within *SGMS1* were in high LD (*r*^2^ = 0.685) but weak LD was observed between rs11006229 and rs10826014 and between rs10826014 and rs12358192 (both *r*^2^ < 0.04) (Supplementary Table S2). Moderate LD was found between rs72759132 and rs4246316 located within *CERS3* (*r*^2^ = 0.26) and between rs6109681 and rs3906631 within *SPTLC3* (*r*^2^ = 0.49). Weak LD was also observed between rs80165332 and rs75397325 located within *CERS6* (*r*^2^ < 0.01).

## 4. Discussion

In recent decades, attention to common chronic diseases, such as obesity, has steadily increased globally. Commonly, MetS is characterized by a collection of these chronic diseases, and, due to the complexity of multiple causes, the clinical features associated with MetS are very different. Furthermore, due to the comprehensive nature of MetS, the causes for specific phenotypes are very diverse. Genetic factors are known to account for the largest proportion of these causes; thus, identification of genetic factors can possibly assist in screening for potential risk factors or in the development of therapeutic materials.

From this perspective, we designed a study to identify genetic factors that can increase blood ceramide levels in Korean subjects with MetS. All clinical indicators except for total cholesterol showed statistically significant differences between MetS group and healthy control (*p* < 5.0 × 10^−^^2^), suggesting that the subjects with MetS who participated in this study have distinct metabolic disorders compared to the controls. The current study evaluated plasma ceramide concentrations as well as SNP genotypes in both subjects with MetS and healthy controls. As with previous studies [27,28], we found ceramide accumulation in the MetS group compared to the control group. Especially, the plasma levels of six ceramide species (Cer-16, Cer-18, Cer-20, DhCer-18, DhCer-24, and DhCer-24:1) reached statistical significance. However, DhCer-16 did not meet the statistical significance criteria, suggesting that the blood dihydroceramide species of participants in this study may not be a risk factor associated with the development of MetS. A recent study has reported that the signals corresponding to 1-deoxyceramides (DxCer) as a non-canonical class of ceramide can be overlapped with those from dihydroceramides in LC-MS/MS spectral analysis [29]. However, we only considered dihydroceramides in this study because enzymes involved in the synthesis of dihydroceramide and ceramide from palmitoyl-CoA and serine as precursors are the same as those involved in the synthesis of DxCer from pamitoyl-CoA and alanine.

Along with these results, our study using the SNP genotyping method has discovered several SNPs for elevated plasma ceramides and dihydroceramides. Previous studies have reported that sphingolipid metabolism is involved in the pathogenesis of many human diseases [30]. The increase in the concentration of sphingolipids, such as ceramide, in the blood is a representative phenotype of MetS. Moreover, increased concentration of ceramide in the blood is known to be a risk factor for the pathogenesis of cancer [31], cardiovascular diseases [10], and age-related disorders such as Alzheimer’s disease and mild cognitive impairment [32,33], which could cause secondary disease due to MetS.

In this study, we identified genetic variants across these genes involved in the ceramide biosynthetic pathway and suggested that these variants affect the functional roles of these genes and are involved in the accumulation of ceramide in the plasma of individuals with MetS. CERS3, CERS4, and CERS6 are members of the ceramide synthase family, which consists of six isoforms (CERS1, CERS2, CERS3, CERS4, CERS5, and CERS6). These ceramide synthase enzymes regulate sphingolipid synthesis by catalyzing the formation of ceramides from sphingoid base and acyl-coA substrates [24], variants of which could have been associated with ceramide synthesis overload. 

ACER1 is an alkaline ceramidase that regulates the levels of ceramides, sphingosine, and sphingosine-1-phosphate by controlling the metabolism of ceramides [25]. Generally, alkaline ceramidase is known to function in the epidermis and plays an important role in skin homeostasis. However, another alkaline ceramidase is known to function in the liver and catalyze the hydrolysis of ceramides [34]. Combining the existing literature data and the results of our work on the association between blood ceramide concentration and genotype, ACER1 could be a candidate that shows correlation with blood ceramide concentration by acting in the liver. SGMS1, also known as SMS1, is a family of sphingomyelin synthases and is known to metabolize ceramide to yield sphingomyelin, which causes a defect in SGMS1 to cause disruption of sphingolipid metabolism [35]. SPTLC2 and SPTLC3, the family of serine palmitoyltransferases, encode a long chain base subunit of serine palmitoyltransferase. Serine palmitoyltransferase is the key enzyme in sphingolipid biosynthesis, and plasma ceramide concentrations are known to be affected by serine palmitoyltransferase activity [36]. 

In general, each of the six CERS isoforms shows a rather specific affinity for certain fatty acids. Therefore, the ceramide *N*-acylation pattern could be related to the underlying activities of the CERS isoforms. In general, CERS3 is specific for ceramide species with very long chain fatty acids such as Cer-26 [37]. Therefore, CERS3 deficiency may result in loss of very long chain ceramides. However, it is not yet clear how specific SNPs at *CERS3* locus of individuals with MetS affect changes in the concentration of long chain ceramides in their plasmas. Interestingly, our results revealed that the *CERS3* is significantly associated with Cer-16 (Table 3), suggesting that the two SNPs (rs72759132 and rs4246316) at *CERS3* locus of individuals with MetS may play a role in increasing the plasma concentration of long chain ceramides such as Cer-16 rather than very long chain ceramides such as Cer-26. However, in this study, we could not identify the effect of the two SNPs on the plasma concentration of Cer-26. In this regard, further research on association between plasma concentrations of more diverse long chain and very long chain ceramides and the two SNPs in the *CERS3* locus identified in our results will be needed. 

SPTLC3 is also known to be involved in the biosynthesis of structurally different long chain ceramides. A study has shown that the overexpression of SPTLC3 results in the biosynthesis of C16-sphingoid bases and ceramides with a C16 backbone account for a significant proportion of sphingolipids in human plasma [38]. In this study, we identified that the two SNPs (rs6109681 and rs3906631) at *SPTLC2* locus may be associated with elevated plasma Cer-16 in individuals with MetS, suggesting that the two SNPs may be associated with the overexpression of SPTLC2 in individuals with MetS. 

Our study also provided information on whether the SNPs within the relevant chromosome regions were in LD with genes involved in sphingolipid biosynthesis in the HapMap CEU, CHB, and JPT data. Interestingly, LD analyzed based on CHB and JPT data showed higher LD than that based on the CEU data, suggesting that LD may vary depending on race and ethnicity.

Hicks and coworkers reported genetic determinants associated with blood sphingolipid concentrations in European populations by a genome-wide association study in 2009 [18], suggesting that SNPs across seven genes involved in ceramide biosynthesis and trafficking, including *SPTLC3*, were associated with ceramide concentrations. However, it was not clear whether the participants that participated in their study had MetS. Nevertheless, their study suggested that blood levels of several important factors involved in sphingolipid metabolism are under genetic control. SNPs identified by Hicks and coworkers did not overlap with those identified by our study, suggesting that SNPs associated with increased blood ceramides may differ between races and ethnicities. Recently, GWAS of Koreans with MetS have reported novel SNPs associated with high TG and low HDL-cholesterolemia [13]. Although these variants are specific to Koreans, the variants are not related to elevated blood ceramides in Koreans with MetS. To date, no studies have reported putative SNPs associated with increased plasma ceramides in Koreans with MetS. Therefore, the novel SNPs may be a genetic predisposition linked to elevated plasma ceramide in Koreans with MetS and a potential therapeutic target for MetS. However, further studies will be needed to uncover the functionalities of these SNPs in a large cohort of MetS.

Although SNPs associated with elevated plasma ceramide levels in Koreans have been identified in our study, a large cohort study to elucidate a detailed role of sphingolipid metabolic pathway genes in the development of MetS using methods such as gene expression analyses will also be needed. 

In conclusion, associations of specific SNPs in the ceramide synthesis genes with six types of ceramides (Cer-16, Cer-18, Cer-20, DhCer-18, DhCer-24, and DhCer-24:1) were identified in individuals with MetS. Further intensive study of the association of these variants and repertoire analyses in different ethnicities and subpopulations could provide insights into more detailed roles of the variants linked to elevated plasma ceramide levels in individuals with MetS.

## Figures and Tables

**Table 1:** Clinical characteristics of study participants.

	Control	MetS	*p* Value
Number of study participants	38	37	
Age (years)	38.9 ± 11.7	46.6 ± 10.3	3.5 × 10^−3^
Height (cm)	165.1 ± 8.1	170.3 ± 8.9	9.9 × 10^−3^
Weight (kg)	58.1 ± 9.7	82.5 ± 16.1	<1.0 × 10^−4^
Waist measurement (cm)	75.2 ± 6.9	94.6 ± 9.7	<1.0 × 10^−4^
BMI (kg/m^3^)	21.2 ± 2.1	27.9 ± 3.4	<1.0 × 10^−4^
SBP (mmHg)	112.5 ± 9.1	126.2 ± 9.7	<1.0 × 10^−4^
DBP (mmHg)	67.1 ± 6.5	76.7 ± 8.1	<1.0 × 10^−4^
Blood glucose (mg/dL)	91.0 ± 5.4	106.5 ± 13.0	<1.0 × 10^−4^
Total cholesterol (mg/dL)	189.5 ± 30.2	202.3 ± 39.5	0.1
HDL-C (mg/dL)	71.4 ± 12.6	48.4 ± 12.8	<1.0 × 10^−4^
LDL-C (mg/dL)	102.9 ± 31.5	116.8 ± 34.3	6.8 × 10^−2^
TG (mg/dL)	81.3 ± 29.0	205.3 ± 102.5	<1.0 × 10^−4^
AST (U/L)	20.2 ± 5.0	31.3 ± 10.2	<1.0 × 10^−4^
ALT (U/L)	16.3 ± 7.1	42.1 ± 21.9	<1.0 × 10^−4^
γ-GTP (U/L)	17.5 ± 8.5	59.8 ± 64.2	<1.0 × 10^−4^
Serum creatinine (mg/dL)	0.8 ± 0.2	0.9 ± 0.2	4.0 × 10^−4^
GFR (mL/min/1.73 m^2^)	101.1 ± 21.7	92.1 ± 13.7	3.5 × 10^−2^
Leptin (pg/mL)	5089.8 ± 4803.1	7866.6 ± 7743.7	6.4 × 10^−2^
Adiponectin (ng/mL)	6464.2 ± 3806.9	2537.2 ± 1633.2	<1.0 × 10^−4^
hs-CRP (mg/L)	0.9 ± 1.3	1.5 ± 1.2	4.0 × 10^−2^
Insulin (µIU/mL)	4.7 ± 2.6	12.4 ± 6.5	<1.0 × 10^−4^
HOMA-IR	1.1 ± 0.6	3.3 ± 1.9	<1.0 × 10^−4^

Values are shown as mean ± SD. BMI, body mass index; SBP, systolic blood pressure; DBP, diastolic blood pressure; HDL-C, high-density lipoprotein-cholesterol; LDL-C, low-density lipoprotein cholesterol; TG, triglycerol; AST, aspartate aminotransferase; ALT, alanine aminotransferase; γ-GTP, γ-glutamyltranspeptidase; GFR, glomerular filtration rate; hs-CRP, high-sensitivity C-reactive protein; HOMA-IR, homeostatic model assessment of insulin resistance.

**Table 2:** The levels of ceramides and dihydroceramides in control and MetS groups.

	Control (ng/mL)	MetS (ng/mL)	*p* Value
Cer-16	61.8 ± 12.7	80.0 ± 17.6	<1.0 × 10^−4^
Cer-18	34.9 ± 12.9	65.2 ± 25.4	<1.0 × 10^−4^
Cer-20	45.3 ± 14.1	79.1 ± 24.9	<1.0 × 10^−4^
DhCer-16	7.6 ± 3.6	8.8 ± 3.4	0.13
DhCer-18	9.9 ± 8.8	18.4 ± 12.4	9.0 × 10^−4^
DhCer-24	122.1 ± 70.7	163.8 ± 60.6	7.2 × 10^−3^
DhCer24:1	99.5 ± 54.0	136.9 ± 62.4	6.6 × 10^−3^

Values are shown as mean ± SD.

**Table 3:** SNPs associated with elevated plasma ceramides.

Gene	dbSNP ID	Chr	Position	Ref ^†^ allele	Alt ^ǂ^ allele	Associated Ceramide	FDR-Adjusted *p* Value
*CERS6*	rs75397325	2	169607910	G	C	Cer-16 Cer-20	5.2 × 10^−3^ 2.1 × 10^−2^
*CERS3*	rs72759132	15	101023194	G	A	Cer-16	5.0 × 10^−2^
*CERS3*	rs4246316	15	101066561	C	T	Cer-16	3.1 × 10^−2^
*CERS6*	rs80165332	2	169516096	C	T	Cer-16	2.8 × 10^−2^
*ACER1*	rs62106618	19	6314542	A	G	DhCer-18 DhCer-24 DhCer-24:1	3.4 × 10^−2^ 4.7 × 10^−2^ 2.6 × 10^−2^
*SGMS1*	rs12358192	10	52364839	T	C	Cer-16 Cer-18 Cer-20	2.6 × 10^−3^ 2.3 × 10^−2^ 1.5 × 10^−2^
*SGMS1*	rs11006229	10	52350006	T	C	DhCer-24 Cer-16 Cer-18 Cer-20	3.9 × 10^−2^ 7.8 × 10^−3^ 1.8 × 10^−2^ 1.0 × 10^−2^
*SGMS1*	rs10826014	10	52209580	T	C	Cer-18	4.2 × 10^−2^
*SPTLC2*	rs149162405	14	78076411	T	C	Cer-20	3.6 × 10^−2^
*SPTLC3*	rs6109681	20	13047504	T	C	Cer-16	4.4 × 10^−2^
*SPTLC3*	rs3906631	20	13064936	C	A	Cer-16	1.3 × 10^−2^

^†^ Ref, reference allele; ^‡^ Alt, alternate allele. Abbreviations: *CERS*, ceramide synthase; *ACER*, alkaline ceramidase; *SGMS*, sphingomyelin synthase; *SPTLC*, serine palmitoyltransferase long chain base subunit.

## Data Availability

Data is available from the corresponding author upon reasonable request.

## Supplementary Materials

The following supporting information can be downloaded at: https://www.mdpi.com/article/10.3390/genes13081497/s1, Table S1: LD analysis of the SNPs located within *CERS3*, *CERS6*, *SGMS1*, and *SPTLC3* genes involved in sphingolipid biosynthesis in the HapMap CEU data. LD was represented by correlation coefficient (*R*^2^); Table S2: LD analysis of the SNPs located within *CERS3*, *CERS6*, *SGMS1*, and *SPTLC3* genes involved in sphingolipid biosynthesis in the HapMap CHB and JPT data. LD was represented by *R*^2^.
