# Supplementary material for: Genetic Variants Associated with Elevated Plasma Ceramides in Individuals with Metabolic Syndrome
Source: Genes (Basel). 2022 Aug 22;13(8):1497. doi: 10.3390/genes13081497 (PMC9407997; doi:10.3390/genes13081497)
Supplement: Supplementary file 1 [file genes-13-01497-s001.zip › genes-1837587-supplementary.pdf]

Supplementary Files

Communication

Genetic variants associated with elevated plasma ceramides in individuals with metabolic syndrome

Sanghoo Lee [1,*], Seol-A Kim [1], Yejin Kim [1], Juhoon Kim [1], Gayeon Hong [1], Jeonghoon Hong [1], Kyeonghwan Choi [2],Chun-Sick Eom [2], Saeyun Baik [3], Mi-Kyeong Lee [4] and Kyoung-Ryul Lee [1,2,3,4,*]

[1] Center for Companion Biomarker, Seoul Clinical Laboratories Healthcare Inc., Yongin 16954, Gyeonggi-do, Korea
[2] Center for Health Check-Up, HANARO Medical Foundation, Seoul 03159, Korea
[3] Central Laboratory, Seoul Clinical Laboratories Healthcare Inc., Yongin 16954, Gyeonggi-do, Korea
[4] Department of MyGenome, Seoul Clinical Laboratories, Yongin 16954, Gyeonggi-do, Korea
* Correspondence: sprout30@scllab.co.kr (S.L.); dkrlee@scllab.co.kr (K.-R.L.)

Supplementary Table S1. LD analysis of the SNPs located within *CERS3*, *CERS6*, *SGMS1*, and *SPTLC3* genes involved in sphingolipid biosynthesis in the HapMap CEU data. LD was represented by correlation coefficient (R^2).

	SGMS1			*CERS6*		*CERS3*		*SPTLC3*	
	rs10826014	rs11006229	rs12358192	rs80165332	rs75397325	rs72759132	rs4246316	rs6109681	rs3906631
SGMS1									
rs10826014	-	0.004	0.009						
rs11006229	0.004	-	0.292						
rs12358192	0.009	0.292	-						
CERS6		-							
rs80165332				-	NA				
rs75397325				NA	-				
CERS3			-						
rs72759132						-	0.137		
rs4246316						0.137	-		
SPTLC3									
rs6109681								-	0.191
rs3906631								0.191	-

NA: not calculated.

Supplementary Table S2. LD analysis of the SNPs located within *CERS3*, *CERS6*, *SGMS1*, and *SPTLC3* genes involved in sphingolipid biosynthesis in the HapMap CHB and JPT data. LD was represented by R^2.

	SGMS1			*CERS6*		*CERS3*		*SPTLC3*	
	rs10826014	rs11006229	rs12358192	rs80165332	rs75397325	rs72759132	rs4246316	rs6109681	rs3906631
SGMS1									
rs10826014	-	0.036	0.008						
rs11006229	0.036	-	0.685						
rs12358192	0.008	0.685	-						
CERS6		-							
rs80165332				-	0.001				
rs75397325				0.001	-				
CERS3			-						
rs72759132						-	0.264		
rs4246316						0.264	-		
SPTLC3									
rs6109681								-	0.490
rs3906631								0.490	-

NA: not calculated.
